# Sleeping on oxidative stress as a mechanism underlying cardiovascular risk in chronic anxiety

**DOI:** 10.1152/ajpheart.00219.2026

**Published:** 2026-03-24

**Authors:** Jeremy A. Bigalke, Nathaniel D. M. Jenkins

**Affiliations:** 1 Department of Health, Human Performance, and Recreation, Baylor University, Waco, Texas, United States; 2 Department of Health, Sport, and Human Physiology, University of Iowa, Iowa City, Iowa, United States; 3 Department of Psychological and Brain Sciences, University of Iowa, Iowa City, Iowa, United States; 4 Abboud Cardiovascular Research Center, University of Iowa, Iowa City, Iowa, United States

Chronic anxiety disorders are increasingly common, with estimates predicting that nearly 30% of individuals in the United States will be diagnosed with an anxiety disorder over the course of their lifetime (1). Perhaps more alarming is the early age of onset, with an estimated 75% of anxiety disorder diagnoses occurring before the age of 21 (1). Anxiety disorders are particularly pernicious because they lack objective biomarkers for characterization or diagnosis, yet are associated with long-term physiological health concerns, including elevated cardiovascular risk. Pooled analyses from cross-sectional and prospective studies demonstrate that chronic anxiety disorder diagnosis is associated with a ~40% increased risk of arterial hypertension (2), which significantly increases the likelihood of subsequent cardiovascular disease progression (3). The high prevalence of anxiety disorders in modern society, paired with the disproportionate cardiovascular burden associated with the onset and progression of anxiety disorders, necessitates the exploration of mechanisms linking chronic anxiety to cardiovascular dysfunction to inform targeted therapeutic interventions.

In this journal, Richardson et al. (4) take an important step toward disentangling the mechanisms underlying the prospective association between chronic anxiety and cardiovascular risk. Using a rigorous, randomized, placebo-controlled, crossover design, the investigators characterized the influence of acute antioxidant supplementation (400 mg α-lipoic acid, 500 mg vitamin C, and 200 IU vitamin E) versus placebo on oxidative stress, vascular function, and blood pressure control in younger adults with chronic generalized anxiety (4). Building upon recent findings from the same research team (5), the authors additionally investigated whether the beneficial effects of antioxidant supplementation might be strongest in adults with chronic anxiety who also displayed greater variability in actigraphy-measured nightly total sleep duration.

Before analyses, the study population was divided using a median split into those with low versus high total sleep duration variability. Importantly, the resultant groups were well-matched across all other demographic variables, mean habitual sleep and physical activity levels, resting hemodynamics, and anxiety/depression symptoms. The authors report that whole blood superoxide production was elevated, whereas flow-mediated dilation and cardiovagal baroreflex sensitivity were reduced, in individuals with high versus low sleep duration variability during the placebo condition (4). Notably, these results were robust to treatment of sleep duration variability as a continuous variable using generalized linear models. Although causal inference cannot be applied, these findings hint at potentiation of oxidative stress and cardiovascular dysfunction in individuals with chronic anxiety and high nightly sleep duration variability. Following antioxidant supplementation, the authors reported that superoxide production was reduced, whereas cardiovagal baroreflex sensitivity was improved only within the high sleep duration variability group. However, flow-mediated dilation was elevated following antioxidant supplementation in both groups, suggesting an indiscriminate beneficial effect of antioxidant supplementation on macrovascular function (4).

The findings of Richardson et al. (4) offer crucial information regarding the mechanistic role of oxidative stress on cardiovascular risk in individuals with chronic anxiety. The findings build upon accumulating evidence that implicates oxidative stress as a mechanistic target underlying disproportionate cardiovascular disease risk across numerous conditions, including exposure to adverse childhood experiences (6), major depressive disorder (7), and posttraumatic stress disorder (8). The coexistence of oxidative stress and cardiovascular dysfunction across etiologically and functionally distinct anxiety, mood, and trauma-related disorders suggests that oxidative stress and subsequent detriments in vascular function may represent a common mechanism underlying cardiovascular disease risk in these populations, and thus a promising transdiagnostic therapeutic target.

We also commend the authors for their novel examination of how objectively quantified habitual sleep duration variability modifies the beneficial cardiovascular effects of antioxidant supplementation. Sleep duration variability has garnered attention as a stronger predictor of cardiovascular risk beyond that which can be gleaned from average sleep duration alone (9). Elevated oxidative stress and reduced vascular and blood pressure control at baseline, alongside pronounced improvements across these variables in the high sleep duration variability group (4), support the potential role of oxidative stress as a mechanistic link between sleep irregularity and cardiovascular disease (9). However, it is difficult to determine the independent influence of chronic anxiety and sleep irregularity on oxidative stress in the absence of a healthy control group. It is interesting to note that the improvements observed in the high sleep duration variability group following antioxidant supplementation reduced superoxide production and improved cardiovascular control, such that they were comparable with the low sleep duration variability group in the placebo condition (4). Whether these improvements remain below the thresholds for oxidative stress, vascular function, and blood pressure control generally observed in a healthy population is an important area for future investigation.

Interestingly, the authors did not observe improvement in resting blood pressure despite improved vascular function and baroreflex sensitivity following antioxidant supplementation. This is an important point as anxiety is robustly associated with elevations in resting blood pressure (10), which significantly increases the risk of cardiovascular disease (3). The study population primarily consisted of healthy, normotensive young adults, which may be one reason underlying the absence of an effect on blood pressure (4). In addition, the acute antioxidant dosing scheme may not have been sufficient to influence long-term blood pressure regulation. Future studies might aim to investigate whether chronic antioxidant supplementation offers beneficial therapeutic effects on blood pressure regulation in individuals with chronic anxiety, particularly in those with elevated blood pressure (10).

In conclusion, Richardson et al. (4) provide critical insights into the mechanistic influence of oxidative stress alongside sleep duration irregularity on subclinical cardiovascular risk markers in younger adults with chronic anxiety. The findings are bolstered by a rigorous study design, objective sleep monitoring, and a well-matched comparison between adults with high versus low sleep irregularity using high-fidelity cardiovascular assessment. The present findings offer a scaffolding for future research to build upon to better understand the complex associations between chronic anxiety, sleep health, oxidative stress, and cardiovascular regulation. Future studies targeting oxidative stress and sleep irregularity (independently or jointly) offer an exciting research avenue that will inform the development of holistic therapeutic interventions to alleviate the cardiovascular burden associated with chronic anxiety.
